# Autologous osteophyte grafting for ankle arthrodesis

**DOI:** 10.1051/sicotj/2022007

**Published:** 2022-04-01

**Authors:** Raden Andri Primadhi, Hendra Gunawan, Sylvia Rachmayati, Hermawan Nagar Rasyid

**Affiliations:** 1 Department of Orthopaedics and Traumatology, Universitas Padjadjaran Medical School/Hasan Sadikin Hospital Jalan Pasteur 38 Bandung 40161 Indonesia; 2 Department of Dermato-Venereology, Universitas Padjadjaran Medical School/Hasan Sadikin Hospital Jalan Pasteur 38 Bandung 40161 Indonesia; 3 Department of Clinical Pathology, Universitas Padjadjaran Medical School/Hasan Sadikin Hospital Jalan Pasteur 38 Bandung 40161 Indonesia

**Keywords:** Osteophyte, Ankle, Arthrodesis, Graft

## Abstract

*Purpose*: Various graft sources had been identified to facilitate gap-filling in ankle arthrodesis procedures with related articular defects. This was a preliminary study with the aim of analyzing the efficacy and feasibility of using autologous osteophyte as a grafting source. *Methods*: Retrospective evaluation of ten patients having ankle arthrodesis procedure using identical anterior approach and plate fixation technique was conducted. Basic anthropometric measurements and underlying disease were recorded. Functional outcome and fusion rate were assessed at a 12-month post-surgery follow-up visit. *Results*: The underlying diseases include primary osteoarthritis (OA), post-traumatic OA, rheumatoid arthritis, and Charcot arthropathy. The patient’s age mean was 56.6 years (range 36–71 years), and BMI varied from 17.9 kg/m^2^ to 29.3 kg/m^2^. Nearly all patients had improved functional outcomes as described by foot and ankle ability measure (FAAM) score and fusion rate as described by modified radiographic union score for tibia (RUST). One patient had failed surgery due to implant failure with diminished protective foot sensory. *Conclusion*: Osteophytes from the distal tibia and talar neck were a viable source of bone graft, especially for ankle arthrodesis using anterior approach among various ages and BMI, in which the surgeons would not need additional incision for graft harvesting.

## Introduction

End-stage ankle arthritis is commonly treated by ankle arthrodesis or ankle arthroplasty. Despite the superior functional outcomes of ankle arthroplasty, ankle arthrodesis remains to be the most common surgical treatment because of its wider availability and a lower rate of complications or revision surgeries [1–3]. However, there is possibly an intraarticular gap or bony irregularities formed at the site of fusion that can cause problems such as delayed fusion, failure of bony apposition, or loss of correction. In order to address this problem, defect-filling bone graft materials were frequently used, including autogenous bone, allogeneic bone, xenograft, and synthetic bone graft [4–6]. Careful graft and fixation planning can minimize postoperative complications of the procedure [7]. Autogenous bone is considered to be the ideal implant due to its compatibility with the recipient site and is more economical than other graft source such as synthetic graft. However, harvesting autologous bone graft may result in donor site morbidity, including hemorrhage, pain, increased surgical time, and stress fracture [8, 9].

Osteophytes are abnormal bony outgrowths that develop mostly at the margins of the articular surfaces related to the development of osteoarthritis. Osteophyte formation is regulated by the same molecular mechanism as a normal bone during embryogenesis, a similar process with endochondral bone formation, although its stimulus origin is abnormal [10]. Several studies have shown that osteophytes express various growth factors, such as insulin-like growth factors, transforming growth factors beta (TGF-β), platelet-derived growth factors, and interleukins 1β and 6 [11–14]. Therefore, osteophytes could be a source of bone grafts not only with an osteoconductive effect but also osteoinductive [10, 11]. From a surgical point of view, osteophytes can be easily harvested during ankle arthrodesis procedures for ankle arthritis.

Clinical application of osteophyte in open-wedge high tibial osteotomy surgery has been reported by Akiyama et al. and shown feasibility [11]. To the best of our knowledge, there is no prior report about the osteophyte application for grafting in any joint arthrodesis cases. In this preliminary study, we report our initial cases of ankle arthrodesis procedures using autologous osteophytes to fill the residual gap after the final cut. The purpose of this study was to discern the clinical effect of osteophytes as a defect-filling graft and to be a basis for future related study, not limited to arthrodesis techniques.

## Material and methods

**Figure 1:**
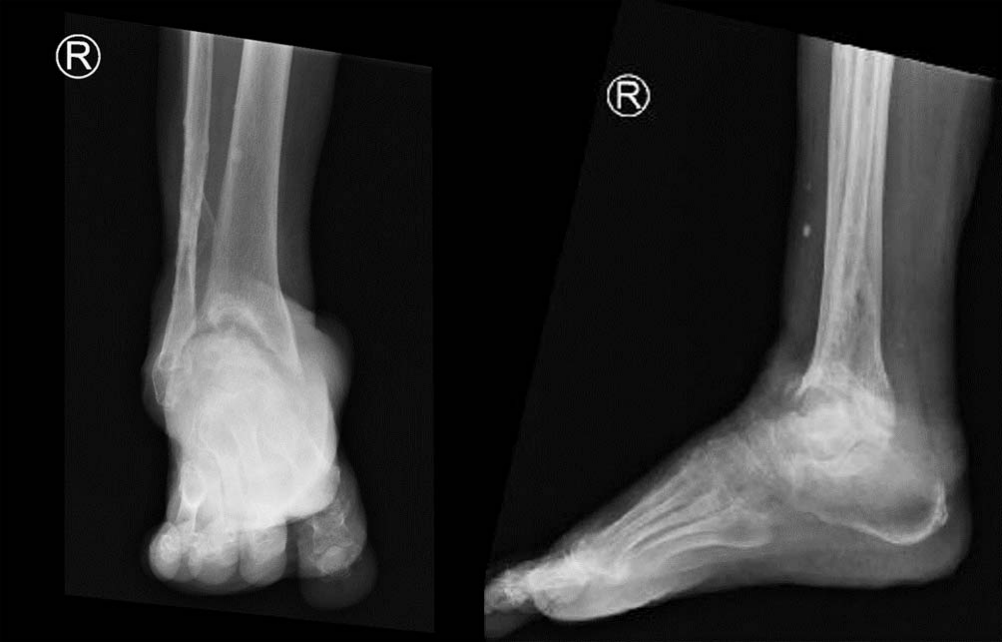
Ankle radiograph showing ankle osteoarthritis with irregular surface that will hamper the bony apposition.

We conducted a retrospective evaluation of 10 consecutive patients (4 male and 6 female) who underwent ankle arthrodesis due to end-stage ankle arthritis with remarkable articular bone loss as illustrated in Figure 1, from January to August 2020 in Hasan Sadikin Hospital, a tertiary referral hospital in Bandung, Indonesia. The patients age’s mean was 56.5 years (range 36–71 years). The underlying diseases were primary ankle osteoarthritis in 3 patients, post-traumatic osteoarthritis in 3 patients, rheumatoid arthritis in 1 patient, post-infection arthritis in 1 patient, and Charcot osteoarthropathy in 2 patients. The patients’ chief complaints included painful weight-bearing, deformity, or instability. Body mass index (BMI) varied from 17.9 kg/m^2^ to 29.3 kg/m^2^.

The patients underwent similar arthrodesis procedures using anterior ankle approach and subsequent fixation with plate and screw (ExtremiLOCK plate, Osteomed, USA). The gap was filled only by autologous osteophytes from the distal tibia or talar neck.

### Operative techniques

**Figure 2:**
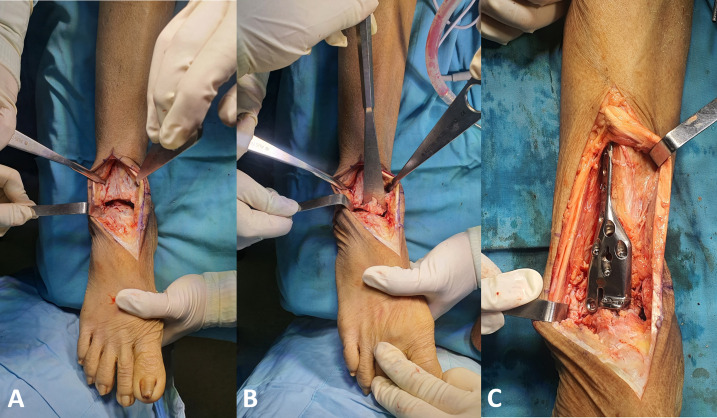
Intraoperative image showing (A) osteophyte formation at distal tibia and talar neck; (B) excision of osteophytes; and (C) final position after osteophyte graft placement and plate fixation.

**Figure 3:**
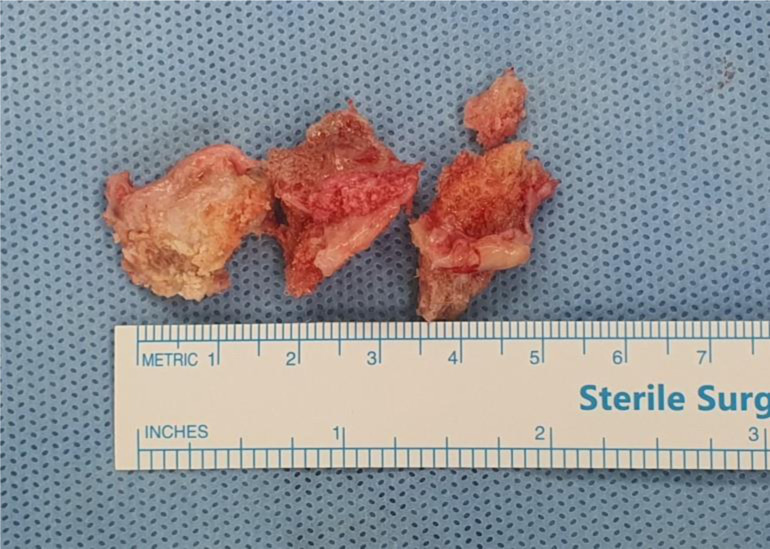
Excised osteophytes from distal tibia and talar neck before morselization.

After standard preparation and draping, ankle arthrodesis procedures were done using the anterior ankle approach for joint denudation, approximation, and fixation. Osteophytes harvesting was done by direct bone cutting using osteotome and chisel during joint preparation (Figures 2A and 2B). Figure 3 shows the autologous osteophyte grafts immediately after being harvested and are morselized with a bone rongeur. After joint preparation and subsequent plate fixation, we inserted the osteophytes into the gap (Figure 2C). All wounds were closed in a standard manner. A sterile bulky dressing and posterior splint were applied.

### Evaluation

Standard anthropometric measurements and clinical functional scores were taken. Preoperative and postoperative radiographs, as well as intraoperative findings, were recorded. The fusion state was analyzed using a modified radiographic union score for tibia (RUST) score taken at a 12-month postoperative follow-up visit. This modified scoring system is derived from the original tibial fracture radiographic union scoring system based on callus formation and visibility of fracture line at four cortices observed on AP and lateral radiographs, introduced by Leow et al. [15]. A minimum score of 4 indicates no healing, and a maximum of 12 indicates a healed fracture. The score for each cortex was assigned according to the criteria shown in Table 1.

**Table 1:** Scoring criteria for modified RUST.

Score per cortex	Bridging callus	Joint gap
1	Absent	Visible
2	Present	Visible
3	Present	Invisible

Outcome scores were measured using Foot and Ankle Ability Measures (FAAM) [16]. The comparison was made between preoperative and 12-month postoperative FAAM scores taken by a single examiner (R.A.P).

Histological examinations were also performed to assess the bone formation potency of the osteophytes from each patient. Specimens of osteophyte grafts were collected and processed to detect any expression of TGF-β inside the graft tissue. TGF-β expression was analyzed using immunohistochemistry staining [17]. Slides were scored based on a system regarding the intensity and extent of the stained area [18, 19] (Table 2).

**Table 2:** Clinical features.

Patient no.	Age (years)	BMI (kg/m^2^)	Disease	Pre-op FAAM Score (%)	12-month post-op FAAM Score (%)	12-month post-op Modified RUST	TGF-β expression
1	62	29.3	Primary OA	25	50	8	+1
2	71	21.5	Charcot osteoarthropathy	19	50	8	+1
3	46	22.0	Rheumatoid arthritis	28	72	8	+1
4	59	23.5	Primary OA	25	56	6	+1
5	61	28.4	Primary OA	38	72	8	+1
6	63	23.7	Post-traumatic OA	19	56	8	+1
7	57	20.8	Post-traumatic OA	28	50	5	+1
8	36	18.7	Post-infection OA	41	56	8	+1
9	47	22.5	Post-traumatic OA	38	72	6	+1
10	63	17.9	Charcot osteoarthropathy	19	N/A	N/A	+1

### Statistical analysis

Statistical analysis of paired sample *t*-test was done using software IBM SPSS Statistics for Windows v. 26.0 (Armonk, NY: IBM Corp.)

## Results

There were improvements in clinical outcomes in nine out of ten patients. The mean FAAM score improved from 28 (range 19–41 points) preoperatively to 59.3 (range 50–72 points) postoperatively. Statistical analysis of paired sample *t*-test revealed significant improvement of FAAM score from preoperative condition (mean score = 28) to 12-month follow-up period (mean 53.4), with *p*-value 0.00135. One patient had to undergo another surgery, specifically transtibial amputation, due to implant failure and subsequent damn nuisance.

**Figure 4:**
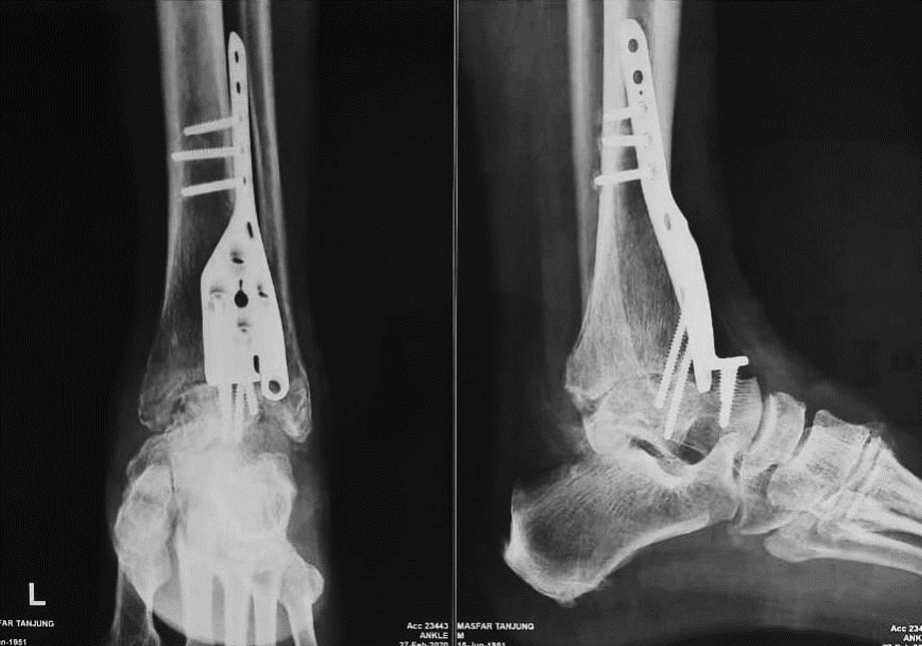
Illustration of modified RUST score (8) consisted of (1) anterior; (2) posterior; (3) lateral; and (4) medial.

Radiological evaluation showed favorable results on bony bridge formation. Modified RUST score analysis at 12-month postoperative follow-up showed slightly different results among patients (ranged from 5 to 8 points) (Figure 4).

**Figure 5:**
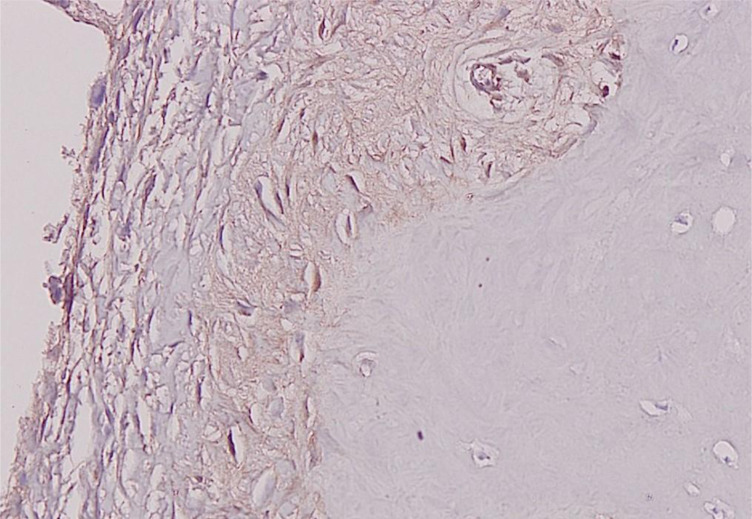
Immunohistochemistry staining result for TGF-β expression from an osteophyte graft, showing score +1 depicting present but weak immunoreactivity.

TGF-β expression as described by cytoplasmic staining was detected in all osteophyte grafts. We used a method by Ding et al., which included assignment of the staining intensity using a scale of 0–10 (with 0 indicating a lack of brown immunoreactivity and 10 reflecting intense dark brown staining) by three observers. The mean was calculated, and the results were converted into grades: 1–3 was assigned “+”, 4–6 was “++”, and more than 7 was “+++” [18, 19]. All osteophyte grafts showed a TGF-β expression score +1 or weak intensity (Figure 5).

## Discussions

Joint fusion demands a proper contact of bone ends. Bony irregularities will need graft as a defect filling material [5]. Numerous graft sources have been reported (Table 3). The investigation is still needed for establishing graft sources that are economical and clinically favorable (i.e., less donor site morbidity). This study reported an advantageous result of using autologous osteophyte as a graft in ankle arthrodesis surgery.

**Table 3:** Prior studies of bone graft source.

No	Year	Authors, journal	Graft source	Remark
1	2005	DeOrio and Farber. FAI [22]	Iliac crest	Minimal morbidity
2	2014	Calori et al. Injury [23]	Iliac crest	Low donor site morbidity
3	2007	Chou et al. FAI [8]	Distal tibia	Stress fracture as complication
4	1999	Alt et al. JOT [24]	Proximal tibia	Low complication rates
5	1998	Biddinger et al. FAI [25]	Calcaneus	Minor complications
6	1996	Hayes and Smith. FAI [26]	Femoral trochanter	Complications: pain, hip discomfort
				Restricted weight bearing
7	1995	Krause and Perry. JOT [27]	Distal femur	Little morbidity
				Nonweight bearing 6 weeks
8	2002	Scranton Jr. FAI [28]	Bone graft substitutes	Successful procedure
9	2016	Akiyama et al. Arthrosc Tech. [11]	Osteophyte from tibial eminence	Easy harvesting. Strong osteoinductive effect
10	2019	Sohn and Oh. Biomater Res [29]	Bone substitute	Single ideal bone graft substitutes still have not been developed

The joint surface preparation should be minimalized to maintain joint congruency. In an incongruent arthritic joint, cutting the bone to make the articular surface congruent is a viable option but, on the other hand, will likely shorten the periarticular bone length resulting in eventual morbidity, for example, limping gait. Ankle fusion using an anteriorly placed plate is proven to be a good method for many types of ankle arthropathies [20]. Considering the presence of a gap on the articular surface and porotic bone, we decided to choose a plate over multiple screws for fixation. Another option to conserve the bone apposition is by minimizing the bone cutting and filling the gap instead with a graft to ensure continuity between the bone ends. Autografts provide a rich source of progenitor cells, bone morphogenetic proteins (BMPs), and growth factors that facilitate new bone formations [14]. Despite its efficacy, autograft harvesting still increases the surgical time and carries donor site morbidity, including infection risk and pain [14, 21]. Allograft and artificial substitute materials are other options to fill the defects. The problems include the paucity and costly features, especially in developing countries. One of the most popular donor sites is the iliac crest, a source of the abundant bone graft [22, 23]. Compared with other graft source such as the femur, tibia, and calcaneus, the iliac crest graft is reported to have less morbidity [8, 24–27]. Despite its low morbidity, there is still a need for an additional incision that prolongs the surgery time and extra preparation. Bone graft substitutes had been introduced for decades with favorable outcomes, although the single ideal one still has not been developed [28, 29].

Osteophytes are osteocartilaginous metaplastic tissues that generally form at the margins of osteoarthritic joints. Active osteophyte growth is a characteristic of osteoarthritis [30, 31]. Osteophyte formation is in addition to joint space narrowing, subchondral sclerosis, and subchondral cyst formation in osteoarthritis. Osteophytes can be a source of pain and loss of function mainly through nerve compression, impingement, limitation of joint mobility, and obstruction of tissues [31]. Pointing osteophytes is also attributable to soft tissue injuries and ruptures. In ankle arthritis, osteophyte is usually formed at the anterior distal tibia and superior aspect of the talar neck resulting in anterior ankle impingement and limitation of joint movement. Isolated anterior ankle impingement is managed by excision of the kissing osteophytes either by arthroscopy, mini-open, or open technique [32]. Although osteophytes can also be present without negative effects or have even positive effects by increasing the joint surface, it is generally accepted that osteophytes are removed during joint arthroplasty and joint arthrodesis to improve access to the joint and to facilitate joint reduction and positioning before implantation or fixation, favorably using the same approach. Histologically, osteophytes are fibrocartilage-capped bony outgrowth originating from the periosteum. Yet, the pathophysiology of osteophyte formation is not completely understood. Osteophytes develop in diarthrodial joints, in which the periosteum and synovial lining contain cells involved in osteophyte formation. Cells in the periosteum are stimulated in a particular condition and subsequently undergo chondrogenesis and deposit matrix molecules, such as aggrecan, in the tissue. Hypertrophy of the chondrocytes is followed by endochondral ossification, deposition of bone, and formation of marrow cavities [31].

The molecular mechanism of osteophyte development has already been well defined. Mesenchymal stem cells within periosteal or synovial tissue differentiate into chondrocytes and form osteophytes via endochondral ossification. Expression of growth factors capable of inducing osteogenesis and chondrogenesis, such as transforming growth factor β (TGF-β) and bone morphogenetic protein-2 (BMP-2) is upregulated during osteophyte formation [14]. Autocrine and paracrine stimulation by TGF-β1 is important in the maintenance and expansion of the mesenchymal stem or progenitor cells of osteoblasts. Bone and cartilage contain large amounts of TGF-β and target cells for TGF-β activity [33]. There is a possibility that these growth factors expressed in osteophytes enhance an anabolic effect on the surrounding osteogenic cells at the graft sites and promote bone healing [11]. TGF-β is a protein superfamily of growth factors regulating cellular activities, including proliferation, differentiation, and tissue migration [34]. TGF-β has a function in maintaining homeostasis of cartilage and bone, extracellular matrix synthesis, angiogenesis, intracellular signaling, and interactions with other growth factors [35]. Osteophytes express a number of growth factors that favor bone union, with the combined effects of these growth factors likely to contribute to osteoblast proliferation, differentiation, and migration [14]. Immunohistochemical analysis is a semi-quantitative analysis to identify the expression of TGF-β in bone samples. In this study, TGF-β expression was found in all osteophytes; however, there were differences in the results among the samples. The data were insufficient to conclude any correlation between TGF-β expression in osteophyte graft and the rate of fusion since the determinant of fusion rate is considered multifactorial.

Considering the quantity of osteophytes, a study by Nagaosa et al. in knee osteoarthritis, the size of osteophytes correlated positively with the severity of local narrowing, local malalignment, and bone attrition [36]. In the ankle joint, that condition that is likely to be found in end-stage ankle arthritis with surface irregularities will predispose the abundant osteophyte formation.

In this case series, we found statistically significant improvement in the FAAM score of the series with the favorable radiological outcome. The most remarkable determinants in improved FAAM were the decreasing pain intensity and the restored ankle stability and alignment. One failed case with Charcot osteoarthropathy (patient no. 10) was attributable to premature ambulation in a less cooperative patient and loss of protective sensory due to peripheral neuropathy. We chose the FAAM score for outcome measurement instead of the more popular American Orthopaedic Foot and Ankle Society (AOFAS) score because all patients in this study had ankle arthrodesis, which will affect the maximum AOFAS score due to one of its features, specifically the sagittal range of motion.

The modified RUST score in this study showed an impression that the fusion rate process advanced favorably. The variation in RUST point was feasibly due to different bone healing capacities in each patient, not on account of surgical or grafting technique. Future studies of the efficacy of osteophyte grafting should include the evaluation of clinical and perioperative features of the patients.

In the treatment of ankle joint osteoarthritis, the anterior approach for ankle arthrodesis has the advantage of completely exposing the joint and facilitating coronal plane deformity correction [37]. Firm fixation is the most important factor for successful fusion. We applied locking plate systems for fixation, as screwing was considered insufficient for incongruent joint arthrodesis with bony defect involving the cortices due to its relative instability.

This result was in concordance with a prior article by Akiyama et al, which assessed osteophytes as a graft source for high tibial osteotomy [11]. In either post-osteotomy fixation or arthrodesis, bone grafts are options to fill any bone defects due to their osteogenic, osteoinductive, and osteoconductive effects, but there are differences in the features in terms of the surgical procedures and anatomical structures. In osteotomy procedures, common indications include deformity correction. The subsequent gap is created in a bone substance and is commonly performed in the vascular supply-rich metaphyseal area. Joint arthrodesis is a procedure to fuse a diseased joint at the end of a bone structure; therefore, if the bony defect is present, a stronger effect of the grafts is required [5].

Obesity was known to be associated with increased rates of postoperative complications but not after ankle arthrodesis [38]. In this study, we found that FAAM scores were improved for all patients, including the ones with high BMI. These results came from short-term follow-up. Longer follow-up is needed in order to better evaluate the clinical outcome of this technique.

The most obvious limitation of this study was the design and the number of cases. This study based on neither a randomized controlled trial nor a comparative study between the use and non-use of bone grafts, or other source of grafts. A prospective, randomized, comparative study involving more cases and a longer follow-up period is considered necessary to verify the validity of these results. Further studies should include other evaluation methods for fusion, such as computed tomography and histology.

Osteophytes harvested locally from the ankle joint were a favorable grafting source from ankle arthrodesis with a bone defect that required gap filling. The advantages of this source of graft included biocompatibility as autologous tissue and surgical advantages as there was no necessity to make separated incisions for graft harvesting.

## Conflict of interest

The authors declare that they have no relevant financial or non-financial interests to report.

## Funding

This research did not receive any specific funding.

## Ethical approval

This study had been approved by the Institutional Review Board of Hasan Sadikin Hospital under protocol No. LB.02.01/X.6.5/6/2020 before commenced.

## Informed consent

Written informed consent was obtained from all patients and/or families.

## Authors contributions

R. A. Primadhi: conceptualization, methodology, investigation, writing original draft, and editing; H. N. Rasyid: investigation, writing, reviewing, and supervision; S. Rachmayati: reviewing, supervision; H. Gunawan: reviewing, supervision.

## References

[1] Terrell RD, Montgomery SR, Pannell WC, Sandlin WC, Inoue H, Wang JC, SooHoo NF (2013) Comparison of practice patterns in total ankle replacement and ankle fusion in the United States. Foot Ankle Int 34, 1486–1492. 23774468 10.1177/1071100713494380

[2] Yasui Y, Hannon CP, Seow D, Kennedy JG (2016) Ankle arthrodesis: A systematic approach and review of the literature. World J Orthop, 7, 700–708. 27900266 10.5312/wjo.v7.i11.700 PMC5112338

[3] Usuelli FG, Indino C, Maccario C, Manzi L, Salini V (2016) Total ankle replacement through a lateral approach: surgical tips. SICOT J 2, 38. 27855774 10.1051/sicotj/2016029 PMC5115059

[4] Jo S-H, Kim Y-K, Choi Y-H (2018) Histological evaluation of the healing process of various bone graft materials after engraftment into the human body. Materials 11, 714. 10.3390/ma11050714 PMC5978091 29724045

[5] Baumhauer JF, Pinzur MS, Daniels TR, Lin SS, Beasley W, Donahue RMJ, DiGiovani CW (2013) Survey on the need for bone graft in foot and ankle fusion surgery. Foot Ankle Int 34, 1629–1633. 23986324 10.1177/1071100713503815

[6] Rosenfeld PF, Budgen SA, Saxby TS (2004) Triple arthrodesis: Is bone grafting necessary? J Bone Joint Surg Br 87-B, 175–178. 10.1302/0301-620x.87b2.15455 15736738

[7] Asomugha EU, Den Hartog BD, Junko JT, Alexander IJ (2016) Tibiotalocalcaneal fusion for severe deformity and bone loss. J Am Acad Orthop Surg 24, 125–134. 26829585 10.5435/JAAOS-D-14-00102

[8] Chou LB, Mann RA, Coughlin MJ, McPeake WT, Mizel MS (2007) Stress fracture as a complication of autogenous bone graft harvest from the distal tibia. Foot Ankle Int 28, 199–201. 17296139 10.3113/FAI.2007.0199

[9] Boone DW (2003) Complications of iliac crest graft and bone grafting alternatives in foot and ankle surgery. Foot Ankle Clin 8, 1–14. 12760571 10.1016/s1083-7515(02)00128-6

[10] Zoricic S, Maric J, Bobinac D, Vukicevic S (2003) Expression of bone morphogenetic proteins and cartilage-derived morphogenetic proteins during osteophyte formation in humans. J Anat 202, 26277. 10.1046/j.1469-7580.2003.00158.x PMC1571079 12713267

[11] Akiyama T, Okazaki K, Mawatari T, Ikemura S, Nakamura S (2016) Autologous osteophyte grafting for open-wedge high tibial osteotomy. Arthrosc Tech 5, e989–e995. 27909665 10.1016/j.eats.2016.04.026 PMC5124027

[12] Dodds RA, Merry K, Littlewood A, Gowen M (1994) Expression of mRNA for IL1 beta, IL6 and TGF beta 1 in developing human bone and cartilage. J Histochem Cytochem 42, 733–744. 8189035 10.1177/42.6.8189035

[13] Horner A, Kemp P, Summers C (1998) Expression and distribution of transforming growth factor-beta isoforms and their signaling receptors in growing human bone. Bone 23, 95–102. 9701467 10.1016/s8756-3282(98)00080-5

[14] Ishihara K, Okazaki K, Akiyama T, Akasaki Y, Nakashima Y (2017) Characterisation of osteophytes as an autologous bone graft source. Bone Joint Res 6, 73–81. 28148490 10.1302/2046-3758.62.BJR-2016-0199.R1 PMC5331175

[15] Leow JM, Clement ND, Tawonsawatruk T, Simpson CJ, Simpson AHRW (2016) The radiographic union scale in tibial (RUST) fractures. Bone Joint Res 5, 116–121. 27073210 10.1302/2046-3758.54.2000628 PMC5009237

[16] Sierevelt IN, Zwiers R, Schats W, Haverkamp D, Terwee CB, Nolte PA, Kerkhoffs GMMJ (2018) Measurement properties of the most commonly used foot- and ankle-specific questionnaires: The FFI, FAOS and FAAM. A systematic review. Knee Surg Sport Traumatol Arthrosc 26, 2059–2073. 10.1007/s00167-017-4748-7 29026933

[17] Yuan S-M, Wang Y-Q, Shen Y, Jing H (2011) Transforming growth factor-β in graft vessels: histology and immunohistochemistry. Clinics 66, 895–901. 21789397 10.1590/S1807-59322011000500029 PMC3109392

[18] Ding W, Zhang W, Hui F, Zhang Y, Zhang F, Li X, Shi F (2012) Cell-specific expression and immunolocalization of nitric oxide synthase isoforms and soluble guanylyl cyclase α1 and β1 subunits in the ovary of fetal, neonatal and immature pigs. Anim Reprod Sci 131, 172–180. 22498451 10.1016/j.anireprosci.2012.02.013

[19] Fedchenko N, Reifenrath J (2014) Different approaches for interpretation and reporting of immunohistochemistry analysis results in the bone tissue – a review. Diagn Pathol 29, 221. 10.1186/s13000-014-0221-9 PMC4260254 25432701

[20] Mohamedean A, Said HG, El-Sharkawi M, El-Adly W, Said GZ (2010) Technique and short-term results of ankle arthrodesis using anterior plating. Int Orthop 34, 833–837. 19763567 10.1007/s00264-009-0872-4 PMC2989010

[21] Fasolis M, Boffano P, Ramieri G (2012) Morbidity associated with anterior iliac crest bone graft. Oral Surg Oral Med Oral Pathol Oral Radiol 114, 586–591. 22901642 10.1016/j.oooo.2012.01.038

[22] DeOrio JK, Farber DC (2005) Morbidity associated with anterior iliac crest bone grafting in foot and ankle surgery. Foot Ankle Int 26, 147–151. 15737257 10.1177/107110070502600206

[23] Calori GM, Colombo M, Mazza EL, Mazzola S, Malagoli E, Mineo GV (2014) Incidence of donor site morbidity following harvesting from iliac crest or RIA graft. Injury 45, S116–S120. 25457330 10.1016/j.injury.2014.10.034

[24] Alt V, Nawab A, Seligson D (1999) Bone grafting from the proximal tibia. J Trauma 47, 555–557. 10498314 10.1097/00005373-199909000-00023

[25] Biddinger KR, Komenda GA, Schon LC, Myerson MS (1998) A new modified technique for harvest of calcaneal bone grafts in surgery on the foot and ankle. Foot Ankle Int 19, 322–326. 9622424 10.1177/107110079801900510

[26] Hayes WR, Smith RW (1998) Trochanteric bone grafts in foot and ankle surgery. Foot Ankle Int 17, 402–405. 10.1177/107110079601700708 8832247

[27] Krause JO, Perry CR (1995) Distal femur as a donor site of autogenous cancellous bone graft. J Orthop Trauma 9, 145–151. 7776035 10.1097/00005131-199504000-00010

[28] Scranton PE (2002) Use of bone graft substitutes in lower extremity reconstructive surgery. Foot Ankle Int 23, 689–692. 12199380 10.1177/107110070202300802

[29] Sohn HS, Oh JK (2019) Review of bone graft and bone substitutes with an emphasis on fracture surgeries. Biomater Res 14, 9. 10.1186/s40824-019-0157-y PMC6417250 30915231

[30] Resnick R, Niwayama G (1995) Degenerative disease in extraspinal locations. In: Diagnosis of Bone and Joint Disorders, 2nd edn. Resnick D, Editor. Philadelphia, WB Saunders.

[31] van der Kraan PM, van den Berg WB (2007) Osteophytes: Relevance and biology. Osteoarthr Cartil 15, 237–244. 10.1016/j.joca.2006.11.006 17204437

[32] Vaseenon T, Amendola A (2012) Update on anterior ankle impingement. Curr Rev Musculoskelet Med 5, 145–150. 22403038 10.1007/s12178-012-9117-z PMC3535150

[33] Chen G, Deng C, Li Y-P (2012) TGF-β and BMP signaling in osteoblast differentiation and bone formation. Int J Biol Sci 8, 271–288. 10.7150/ijbs.2929 PMC3269610 22298955

[34] Einhorn TA, Gerstenfeld LC (2015) Fracture healing: Mechanism and intervention. Nat Rev Rheumatol 11, 45–54. 25266456 10.1038/nrrheum.2014.164 PMC4464690

[35] Patil AS, Sable RB, Kothari RM (2011) An update on transforming growth factor-B (TGF-B): Sources, types, functions and clinical applicability for cartilage/bone healing. J Cell Physiol 226, 3094–3103. 21344394 10.1002/jcp.22698

[36] Nagaosa Y, Lanyon P, Doherty M (2002) Characterisation of size and direction of osteophyte in knee osteoarthritis: A radiographic study. Ann Rheum Dis 61, 319–324. 11874834 10.1136/ard.61.4.319 PMC1754047

[37] Kim J-G, Ha D-J, Gwak H-C, Kim C-W, Kim J-H, Lee S-J, Kim Y-J, Lee C-R, Park J-H (2018) Ankle arthrodesis: A comparison of anterior approach and transfibular approach. Clin Orthop Surg 10, 368–373. 30174814 10.4055/cios.2018.10.3.368 PMC6107825

[38] Kamalapathy PN, Plessis MID, Chen D, Bell J, Park JS, Werner BC (2021) Obesity and postoperative complications following ankle arthrodesis: A propensity score matched analysis. J Foot Ankle Surg 60, 1193–1197. 34127372 10.1053/j.jfas.2021.05.004

